# Clinical implications of an intraoperative margin-positive distal bile duct in perihilar cholangiocarcinoma: international multicentre cohort study

**DOI:** 10.1093/bjsopen/zrag010

**Published:** 2026-03-23

**Authors:** Hasan Ahmad Al-Saffar, Britte H E A ten Haaft, Rebecca Marino, Mario De Bellis, Pim B Olthof, Sondre Busund, Nicolai A Schultz, Stefan Gilg, Andrea Ruzzenente, Bas Groot Koerkamp, Joris I Erdmann, Francesca Ratti, Sheraz Yaqub, Hans-Christian Pommergaard, Christian Sturesson

**Affiliations:** Department of Digestive Diseases, Transplantation and General Surgery, Centre for Cancer and Organ Diseases, Copenhagen University Hospital, Rigshospitalet, Copenhagen, Denmark; Hepatic Malignancy Surgical Research Unit (HEPSURU), Copenhagen University Hospital, Rigshospitalet, Copenhagen, Denmark; Division of Surgery and Oncology, Department of Clinical Science, Intervention and Technology, Karolinska Institutet and Karolinska University Hospital, Stockholm, Sweden; Department of Surgery, Amsterdam UMC, University of Amsterdam, Amsterdam, the Netherlands; Cancer Center Amsterdam, Amsterdam, The Netherlands; Hepatobiliary Surgery Division, IRCCS Ospedale San Raffaele, Milano, Italy; Division of General and Hepatobiliary Surgery, Department of Surgery, Dentistry, Paediatrics and Gynaecology, G. B. Rossi Hospital, University of Verona, Verona, Italy; Department of Surgery, Amsterdam UMC, University of Amsterdam, Amsterdam, the Netherlands; Department of Surgery, Erasmus MC University Medical Center, Rotterdam, the Netherlands; Department of Hepatopancreatobiliary Surgery, Oslo University Hospital, University of Oslo, Oslo, Norway; Department of Digestive Diseases, Transplantation and General Surgery, Centre for Cancer and Organ Diseases, Copenhagen University Hospital, Rigshospitalet, Copenhagen, Denmark; Division of Surgery and Oncology, Department of Clinical Science, Intervention and Technology, Karolinska Institutet and Karolinska University Hospital, Stockholm, Sweden; Division of General and Hepatobiliary Surgery, Department of Surgery, Dentistry, Paediatrics and Gynaecology, G. B. Rossi Hospital, University of Verona, Verona, Italy; Department of Surgery, Erasmus MC University Medical Center, Rotterdam, the Netherlands; Department of Surgery, Amsterdam UMC, University of Amsterdam, Amsterdam, the Netherlands; Cancer Center Amsterdam, Amsterdam, The Netherlands; Hepatobiliary Surgery Division, IRCCS Ospedale San Raffaele, Milano, Italy; Department of Hepatopancreatobiliary Surgery, Oslo University Hospital, University of Oslo, Oslo, Norway; Department of Digestive Diseases, Transplantation and General Surgery, Centre for Cancer and Organ Diseases, Copenhagen University Hospital, Rigshospitalet, Copenhagen, Denmark; Hepatic Malignancy Surgical Research Unit (HEPSURU), Copenhagen University Hospital, Rigshospitalet, Copenhagen, Denmark; Department of Clinical Medicine, University of Copenhagen, Copenhagen, Denmark; Division of Surgery and Oncology, Department of Clinical Science, Intervention and Technology, Karolinska Institutet and Karolinska University Hospital, Stockholm, Sweden

**Keywords:** pancreatoduodenectomy, hepatectomy, biliary tract, R0, frozen section, cancer

## Abstract

**Background:**

The short- and long-term outcomes of intraoperative extension of the distal bile duct margin following a positive frozen section in patients with perihilar cholangiocarcinoma remain poorly characterized.

**Methods:**

Between 1 January 2003 and 1 January 2023, consecutive patients at seven high-volume European hepatopancreatobiliary centres undergoing major hepatectomy for perihilar cholangiocarcinoma with frozen-section analysis of the distal bile duct and subsequent intraoperative management were included retrospectively. The primary endpoint was achievement of an overall R0 resection following distal bile duct re-resection or additional pancreatoduodenectomy after identification of a positive distal bile duct frozen section. Secondary endpoints included overall survival, disease-free survival, and major postoperative complications (Clavien–Dindo grade ≥ IIIa). Outcomes were reported as adjusted hazard ratios.

**Results:**

Of 785 patients undergoing major hepatectomy for perihilar cholangiocarcinoma, 594 underwent distal bile duct frozen-section analysis. A total of 66 (11.1%) intraoperative frozen sections were positive for invasive carcinoma, with an additional 7 (1.2%) false-negative findings. Distal bile duct re-resection was performed in 49 patients (74%), and 11 (16%) underwent additional pancreatoduodenectomy. Among these, 46 patients (69%) achieved distal bile duct margin clearance, with 30 (45%) ultimately attaining overall R0 resection. Overall survival was similar for patients who achieved overall R0 status via distal bile duct re-resection or pancreatoduodenectomy and those with overall R0 margins after primary negative distal bile duct frozen section (adjusted hazard ratio 0.84, 95% confidence interval 0.49 to 1.44; *P* = 0.536). Conversely, overall R1 resection was associated with significantly worse overall survival (adjusted hazard ratio 1.82, 1.43 to 2.33; *P* < 0.001). Complications graded ≥ IIIa and 90-day mortality did not differ significantly between groups undergoing distal bile duct re-resection, pancreatoduodenectomy or no intervention (*P* = 0.126 and *P* = 0.121, respectively).

**Conclusion:**

In selected patients, re-resection or additional pancreatoduodenectomy after positive distal bile duct frozen-section analysis is associated with long-term survival without significantly increasing major morbidity or mortality.

## Teaser

In this multicentre cohort of 785 patients with perihilar cholangiocarcinoma, 11.1% had a positive distal bile duct frozen section. Re-resection or pancreatoduodenectomy achieved margin clearance in most patients without increasing major morbidity or mortality rates. Long-term survival was comparable to that among patients with primary negative margins, whereas R1 resections carried significantly worse outcomes.

## Introduction

Perihilar cholangiocarcinoma (pCCA) is a challenging malignancy with 5-year overall survival (OS) rates of between 38 and 47% following surgery with curative intent^1–3^. Few patients are eligible for curative resection because of poor clinical performance status, anatomical unresectability or metastatic disease at diagnosis^1,4^. Achieving a radical resection (R0) is considered crucial for long-term survival in surgically treated patients^4^. In most patients, R0 resection necessitates major hepatectomy with extrahepatic bile duct (EHBD) resection, and occasionally vascular resection and reconstruction^4^.

To ensure longitudinal margin clearance during surgery, frozen-section (FS) analysis of both proximal (PBD) and distal (DBD) bile duct margins is undertaken routinely^5^. If a positive margin (R1) is identified in the FS, intraoperative re-resection can be considered to achieve an R0 margin^6^. Extension of the PBD margin is often limited by the extent of liver that can be resected technically while leaving a sufficient liver remnant^5^. A recent European multicentre study^7^ also demonstrated no long-term benefit of PBD margin extension on positive FS.

In contrast, DBD re-resection into the cranial pancreatic head is not considered technically difficult, and harbours fewer technical difficulties and risks. Where margin involvement extends into the deep intrapancreatic portion of the DBD, an additional pancreatoduodenectomy (PD) may be performed to remove the whole DBD segment^8^.

Despite the utility of DBD re-resection, its oncological value has not been well studied, and data on associated postoperative morbidity remain unknown. Existing scarce evidence is based on small single-centre series^8–10^ from Eastern institutions, or studies^11^ that analysed the PBD and DBD together. Furthermore, the benefits of additional PD following a positive DBD FS remain unclear^10^, with some studies^12,13^ reporting postoperative mortality rates as high as 18%.

Given the lack of large-scale multicentre data, clarifying the oncological impact and perioperative risks of DBD re-resection or additional PD is critical for surgical decision-making. This international, multicentre cohort study aimed to investigate both short- and long-term outcomes in patients with resected pCCA, who underwent DBD re-resection or additional PD following a positive DBD FS.

## Methods

Patients (aged ≥ 18 years) with histologically confirmed pCCA who underwent resection with curative intent between 1 January 2003 and 1 January 2023 were included from seven high-volume European hepatopancreatobiliary (HPB) centres: Karolinska University Hospital (Sweden), Copenhagen University Hospital (Denmark), Oslo University Hospital (Norway), IRCCS Ospedale San Raffaele Milano (Italy), G. B. Rossi University Hospital Verona (Italy), Amsterdam UMC (the Netherlands), and Erasmus UMC Rotterdam (the Netherlands). Retrospective data were collected from electronic medical records, including baseline characteristics, surgical and pathological reports, recurrence data, and survival status. All data were anonymized and collected in a standardized data file between January and April 2025. A minimum of 2 years of follow-up was required to ensure assessment of recurrence^14^. Owing to variable data availability, some centres contributed patients from an individual consecutive subset of the inclusion period. Exclusion criteria were: absence of DBD FS, patients not undergoing major hepatectomy, liver transplantation, concomitant PD without previous intraoperative DBD FS, R2 resection, and missing recurrence or DBD margin data. Variations in preoperative and postoperative work-up were accepted given the retrospective nature of the study, but were addressed by including only centres carrying out at least seven resections per year^15,16^. Ethical approval was obtained from the Danish Health Authority in the Capital Region of Denmark (R-23057752), the Regional Ethics Board in Stockholm (2022-06962-02), and the Institutional Review Board at Oslo University Hospital (25/02945). Informed consent was waived owing to the retrospective design. The study adhered to the STROBE guidelines^17^.

### Definitions

The preoperative physical status of included patients was categorized according to the American Society of Anesthesiologists (AS) Physical Status classification^18^. All patients underwent major hepatectomy (≥ 3 Couinaud segments) with EHBD resection^6^. Regional lymphadenectomy was performed routinely. Intraoperative FS were taken from the DBD and assessed in real time by a pathologist followed by direct feedback to the surgeon. The DBD FS was classified as negative (no invasive carcinoma) or positive (presence of invasive carcinoma)^19^. Cases of high-grade dysplasia (HGD) were classified as negative, given the lack of consensus on its clinical relevance during the study period^20,21^. When the DBD FS was positive, DBD re-resection or PD was undertaken based on tumour extent at the surgeon’s discretion. If the final pathological results revealed invasive carcinoma despite a negative intraoperative DBD FS, the result was labelled false-negative; in the reverse situation, the result was labelled false-positive. Tumours were staged in accordance with the American Joint Committee of Cancer 8th edition^22^. Pathological evaluation was undertaken locally at each centre by expert HPB pathologists using International Collaboration on Cancer Reporting (ICCR) criteria^23^, including evaluation of the DBD, PBD, and radial margins (RMs)^24–27^. Overall R0 was defined by ≥ 1 mm clearance at all resection margins and dissection planes; and overall R1 as < 1 mm clearance at one or more resection margin or dissection plane. Because of the retrospective and multicentre nature of the study, central re-review of pathology slides was not feasible.

### Outcomes

The primary outcome was the proportion of patients achieving an overall R0 margin following DBD FS or PD. Secondary outcomes included OS, disease-free survival (DFS), local recurrence, major morbidity (Clavien–Dindo grade ≥ IIIa within 90 days)^28^, postoperative significant bile leakage (International Study Group of Liver Surgery (ISGLS) grade B or C)^29^, postoperative intra-abdominal abscess requiring drainage, and 90-day mortality.

### Statistical analysis

Statistical analyses were undertaken using R 3.5.3 (R Foundation for Statistical Computing, Vienna, Austria). Demographic and clinicopathological variables are documented with missing data. Categorical variables are reported as numbers and percentages, and continuous variables as median (interquartile range).

Clinicopathological variables were described and categorized into three groups to assess long-term outcomes of the DBD FS, with respective *P* values: overall R0 after primary DBD FS, overall R0 after DBD re-resection or additional PD, and overall R1. The overall R1 group included all patients with a final R1 status, irrespective of whether they underwent DBD re-resection, additional PD, or neither. Group differences were assessed using Pearson’s χ^2^ test for categorical data, and the Kruskal–Wallis test for continuous data.

Median follow-up was estimated using the reverse Kaplan–Meier method, from the date of surgery to death from any cause, loss to follow-up, or the end of the study (1 January 2025). OS was calculated from the date of surgery to date of death or last follow-up. DFS was calculated from date of surgery to date of radiologically confirmed recurrence or death.

Univariable and multivariable Cox regression models were used to identify predictors of OS and DFS, in accordance with the REMARK checklist^30^. Known prognostic factors^31–33^ and variables with *P* < 0.100 in the univariable analysis were included in the multivariable analysis. Results from the DBD margin, PBD margin, and RM were reported separately^24–27^, but censored from the analysis to avoid multicollinearity. Assumptions governing the Cox proportional hazards ratio (HR) were assessed graphically and tested using scaled Schoenfeld residuals in the cox.zph function of the R survival package. Of note, logistic regression analysis for 90-day mortality, Clavien–Dindo grade ≥ IIIa complications, bile leakage, and intra-abdominal abscess was not part of the initial study design but was performed *post hoc*; known prognostic factors^31–33^ or those with *P* < 0.200 in univariable analyses were examined in multivariable analysis. Missing data were handled through multiple imputation (mice package for R 3.5.1; 20 imputed data sets, 10 iterations). Survival differences between groups were tested using Kaplan–Meier curves and log rank tests. All *P* values were two-sided, and significance was set at *P* < 0.050.

## Results

### Baseline characteristics

**Fig. 1:**
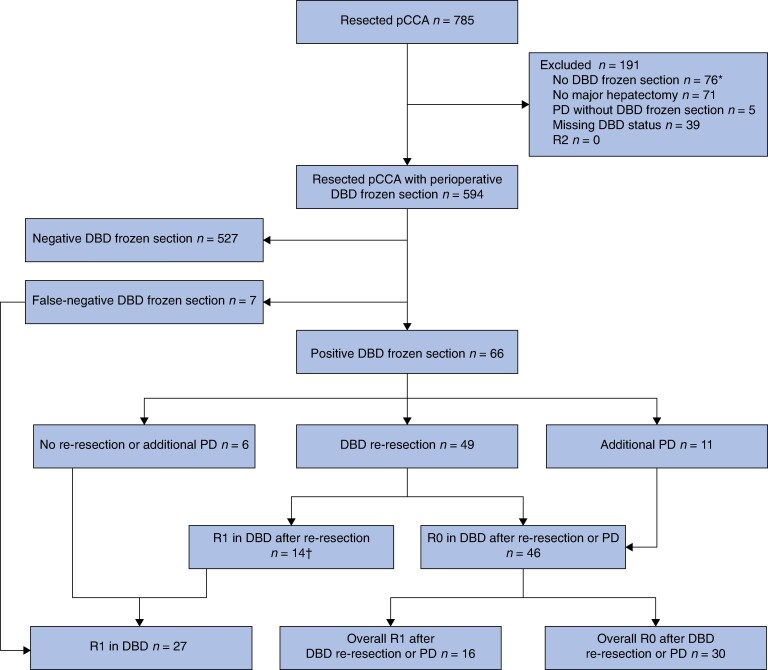
Study flow diagram *Reasons for omitting distal bile duct (DBD) frozen section (FS) analysis are presented in the section entitled Outcomes of positive DBD FS. †One patient had a false-negative DBD re-resection. pCCA, perihilar cholangiocarcinoma; PD, pancreatoduodenectomy.

A total of 785 patients underwent resection for pCCA. The final cohort included 594 patients who underwent intraoperative DBD FS (*Fig. 1*). Of these, 66 (11.1%) had a positive DBD margin during surgery, and 7 (1.2%) were found to have false-negative results. An additional four patients (0.7%) had HGD on DBD FS. No false-positives were reported. Preoperative features of all patients who underwent DBD FS analysis are presented at *Table 1.*

**Table 1:** Preoperative features of all patients who underwent DBD FS analysis

	Pathological outcome following DBD FS			*P*†
	Overall R1 (*n* = 309)*	Overall R0 after re-resection or PD (*n* = 30)	Overall R0 after primary DBD FS (*n* = 255)	
**Age (years), median (i.q.r.)**	67.4 (58.7–73.0)	67.2 (57.1–71.8)	67.2 (59.8–73.4)	0.650‡
Missing	0	0	0	
**Sex**				0.790†
Female	116 (37.5%)	13 (43%)	100 (39.2%)	
Male	193 (62.5%)	17 (57%)	155 (60.8%)	
Missing	0	0	0	
**Year of resection**				0.017†
2003–2009	20 (6.5%)	1 (3%)	29 (11.4%)	
2010–2016	131 (42.4%)	10 (33%)	78 (30.6%)	
2017–2023	158 (51.1%)	19 (64%)	148 (58.0%)	
**ASA fitness grade**				0.734†
I	78 (25.2%)	6 (20%)	57 (22.4%)	
II	167 (54.1%)	15 (50%)	143 (56.1%)	
III	64 (20.7%)	9 (30%)	55 (21.6%)	
**BMI (kg/m^2^), median (i.q.r.)**	24.0 (22.1–26.5)	23.9 (21.7–26.4)	24.8 (22.2–26.8)	0.405‡
Missing	29	1	25	
**Jaundice at presentation**	206 (67.1%)	21 (70%)	184 (72.2%)	0.318†
Missing	2	0	0	
**CA19-9 (U/mL), median (i.q.r.)**	148 (46–546)	84 (59–247)	126 (39–420)	0.900‡
Missing	81	2	48	
**Bismuth–Corlette type**				< 0.001†
I	6 (1.9%)	3 (10%)	7 (2.7%)	
II	24 (7.8%)	7 (23%)	39 (15.3%)	
IIIa	115 (37.2%)	12 (40%)	68 (26.7%)	
IIIb	69 (22.3%)	4 (13%)	86 (33.7%)	
IV	95 (30.8%)	4 (13%)	55 (21.6%)	
Missing	0	0	0	
**Preoperative biliary drainage**				< 0.001†
ERCP	90 (29.1%)	10 (33%)	74 (29.0%)	
PTC	129 (41.7%)	15 (50%)	89 (34.9%)	
Both	44 (14.2%)	2 (7%)	24 (9.4%)	
None	46 (14.9%)	3 (10%)	68 (26.7%)	
Missing	0	0	0	
**Portal vein embolization**	75 (24.3%)	7 (23%)	40 (15.7%)	0.039†
Missing	0	0	0	
**Operation type**				0.009†
Left-sided hemihepatectomy	137 (44.3%)	10 (33%)	142 (55.7%)	
Right-sided hemihepatectomy	163 (52.8%)	20 (67%)	111 (43.5%)	
Central hepatectomy	9 (2.9%)	0 (0%)	2 (0.8%)	
Missing	0	0	0	
**Vascular reconstruction**	77 (24.9%)	8 (27%)	44 (17.3%)	0.071†
Missing	0	0	0	

Values are *n* (%) unless otherwise stated. *Including one patient who had a false-negative intraoperative frozen section (FS). DBD, distal bile duct; PD, pancreatoduodenectomy; i.q.r., interquartile range; ASA, American Society of Anesthesiologists; BMI, body mass index; CA, carbohydrate antigen; ERCP, endoscopic retrograde cholangiopancreatography; PTC, percutaneous transhepatic cholangiography. †χ^2^ test, except ‡Kruskal–Wallis test.

### Outcomes of positive DBD FS

Among the 66 patients with a positive intraoperative DBD FS, 49 underwent re-resection of the DBD, and 11 had additional PD. Six patients received no further surgical intervention after the primary positive DBD FS, and 14 who underwent DBD re-resection with secondary positive DBD FS did not proceed to additional PD. Reasons for not performing DBD re-resection or additional PD included frailty (13), a positive PBD FS (6) or false-negative DBD re-resection (1).

Although 46 of the 66 patients achieved R0 at the DBD after re-resection or additional PD, only 30 (45%) had an overall R0 margin on the final pathology because of remaining R1 margins at the PBD, RM, or both. Of note, 31 patients (47%) with a positive DBD FS had concurrent involvement of the PBD margin and/or RM (*Table 2*); among these, DBD re-resection was performed in 22, and additional PD in 4. All patients undergoing additional PD whose margin status remained R1 had RM involvement only (3), or RM involvement with false-negative PBD FS (1). In four of seven patients with a false-negative DBD FS, there was concurrent involvement of the PBD margin and/or RM.

**Table 2:** Postoperative clinicopathological features of all patients who underwent DBD FS

	Pathological outcome following DBD FS			*P*¶
	Overall R1 (*n* = 309)*	Overall R0 after re-resection or PD (*n* = 30)	Overall R0 after primary DBD FS (*n* = 255)	
**Pathological tumour category**				0.024¶
pT1	39 (12.7%)	0 (0%)	26 (10.3%)	
pT2	137 (44.8%)	18 (60%)	128 (50.8%)	
pT3	96 (31.4%)	11 (37%)	86 (34.1%)	
pT4	34 (11.1%)	1 (3%)	12 (4.8%)	
Missing	3	0	3	
**Lymph node metastasis**	143 (46%)	16 (53%)	103 (40.4%)	0.217¶
Missing	0	0	0	
**False-positive PBD FS**	12			
Positive DBD with re-resection	0			
Positive DBD without re-resection or PD	1			
Negative DBD	11			
Missing	0			
**DBD margin R1**†	27			
DBD re-resection	14 (52%)			
Positive DBD without re-resection or PD	13 (48%)			
Missing	0			
**PBD margin R1**‡	91			
DBD re-resection	6			
Additional PD	1			
Positive DBD without re-resection or PD	6			
Negative DBD	78			
Missing	32§			
**RM R1**	244			
DBD re-resection	12			
Additional PD	4			
Positive DBD without re-resection or PD	8			
Negative DBD	220			
Missing	47§			
**Perineural invasion**	257 (87.1%)	22 (79%)	198 (80.8%)	0.221¶
Missing	14	2	10	
**Tumour differentiation**				0.243¶
Well	38 (14.4%)	1 (4%)	41 (17.4%)	
Moderate	157 (59.5%)	18 (75%)	127 (54.0%)	
Poor	69 (26.1%)	5 (21%)	68 (28.9%)	
Missing	45	6	20	
**Tumour size (mm), median (i.q.r.)**	28 (20–40)	20 (17–28)	21 (16–30)	< 0.001#
Missing	45	1	18	
**Adjuvant chemotherapy**	135 (43.4%)	5 (17%)	63 (24.7%)	< 0.001¶
Missing	0	0	0	
**90-day mortality**	36 (11.7%)	0 (0%)	19 (7.5%)	0.013¶
Missing	0	0	0	
**Recurrence**	171 (55.3%)	17 (57%)	116 (45.5%)	0.054¶
Missing	0	0	0	
**Recurrence location**				< 0.001¶
Local	69 (40.4%)	3 (18%)	28 (24.1%)	
Liver	26 (15.2%)	3 (18%)	30 (25.9%)	
Distant	38 (22.2%)	11 (65%)	39 (33.6%)	
Multifocal	29 (17.0%)	1 (6%)	13 (11.2%)	
Unknown	9 (5.3%)	0 (0%)	6 (5.2%)	

Values are *n* (%) unless otherwise stated. *Including patients who had a false-negative intraoperative frozen section (FS). †Final pathological result for patients with DBD FS with re-resection. ‡Final pathological result for patients with DBD or proximal bile duct (PBD) FS with re-resection or pancreatoduodenectomy (PD). §No patient with a positive FS had missing final margin data at the PBD margin or radial margin (RM); 24 patients had all margins evaluated with known overall R status, but individual data were missing for either the PBD margin or RM; data for the PBD margin and RM were missing for 8 and 23 patients, respectively. i.q.r., Interquartile range. ¶χ^2^ test, except #Kruskal–Wallis test.

### Clinicopathological features

Overall R1 resection was associated with a higher T category (*P* = 0.024) and larger tumours (*P* < 0.001), but not with other adverse pathological features. The 90-day mortality rate was also higher in the overall R1 group (11.7%) compared with the groups with either overall R0 after primary DBD FS (7.5%) or overall R0 after DBD re-resection or additional PD (0%) (*P* = 0.013), although pairwise comparison between overall R1 and overall R0 after primary DBD FS was not significant (*P* = 0.051). Recurrence in general did not differ between all groups (*P* = 0.054). Local recurrence was more frequent in the overall R1 group compared with both overall R0 groups (*P* < 0.001). Adjuvant chemotherapy was administered more frequently in the overall R1 group (*P* < 0.001). Pathological outcomes following DBD FS varied across eras (*P* = 0.017).

### Survival analysis

Overall R0 was achieved in 51.5% of the cohort. Median OS was longer in patients with overall R0 resections than in those with overall R1 resections (47.1 (95% confidence interval (c.i.) 26.6 to 99.9) *versus* 28.1 (14.5 to 67.8) months, respectively), with an adjusted HR of 1.83 (95% c.i. 1.44 to 2.32; *P* < 0.001) (*Fig. S1*).

**Fig. 2:**
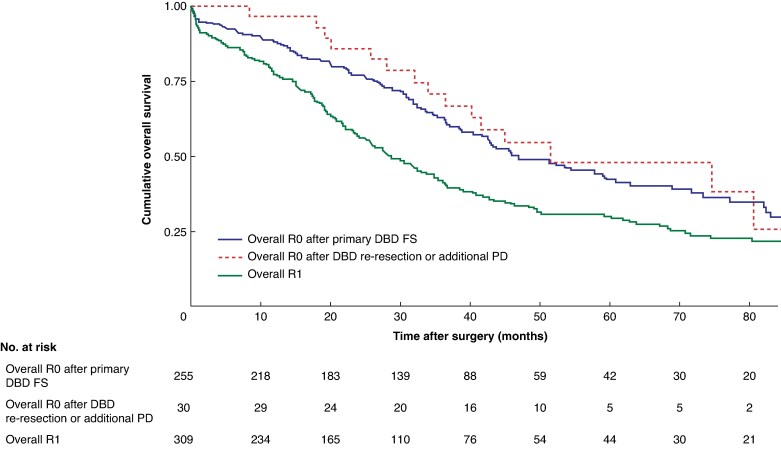
Kaplan–Meier curve showing overall survival according to the outcome of DBD FS analysis Patients were grouped as overall R0 after primary DBD FS, overall R0 after DBD re-resection or additional PD, and overall R1. Pairwise comparisons: *P* = 0.706 (overall R0 after primary DBD FS *versus* overall R0 after DBD re-resection or PD); *P* < 0.001 (overall R0 after primary DBD FS *versus* overall R1); *P* = 0.021 (overall R0 after DBD re-resection or additional PD *versus* overall R1) (log rank test).

Survival by DBD FS outcome is shown in *Fig. 2*. Median OS was 45.5 (26.1 to 87.2) months for overall R0 after primary DBD FS, 51.2 (31.2 to 80.4) months for overall R0 after DBD re-resection or additional PD, and 28.1 (14.5 to 67.8) months for overall R1. There was no survival difference between the two overall R0 groups (*P* = 0.706), but both had longer survival than the overall R1 group (*P* < 0.001 and *P* = 0.021, respectively). Among patients who survived beyond 90 days after surgery, survival remained comparable between the two overall R0 groups (*P* = 0.968) (*File 2*, *Fig. S1*). In contrast to overall R0 after primary DBD FS *versus* overall R1, no difference was observed between overall R0 after DBD re-resection or additional PD and overall R1 (*P* < 0.001 and *P* = 0.071, respectively). Such a relationship was not observed for DFS (*Fig. S3*). A separate analysis with HGD regarded as R1 is shown in *Fig. S4*.

**Fig. 3:**
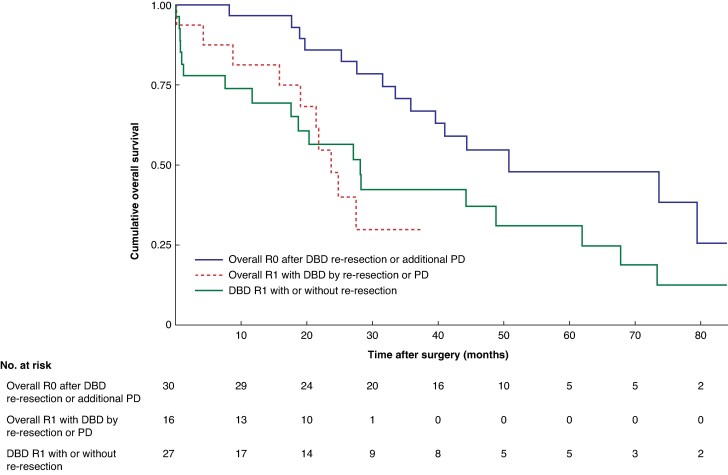
Kaplan–Meier curve showing overall survival according to outcome of positive DBD frozen section Patients were grouped as overall R0 after DBD re-resection or PD, overall R1 with DBD R0 by re-resection or PD, and DBD R1 with or without re-resection. Pairwise comparisons: *P* = 0.002 (overall R0 after DBD re-resection or PD *versus* overall R1 after DBD re-resection or PD); *P* = 0.032 (overall R0 after DBD re-resection or PD *versus* overall R1); *P* = 0.684 (overall R1 after DBD re-resection or PD *versus* overall R1) (log rank test).

Survival for patients with a positive DBD FS is shown in *Fig. 3*. Median OS was 51.2 (31.2 to 80.4) months for overall R0 after DBD re-resection or PD, 23.3 (17.4 to not reached) months for overall R1 with DBD R0 by DBD re-resection or PD, and 27.5 (6.4 to 61.3) months for DBD R1 with or without re-resection. Survival was similar between the two R1 groups (*P* = 0.684), and both had worse survival compared with overall R0 after DBD re-resection or PD (*P* = 0.032 and *P* = 0.002, respectively).

The results of univariable and multivariable Cox regression analyses for OS are shown in *Table 3*. Compared with overall R0 after primary DBD FS, overall R0 after DBD re-resection or additional PD was not associated with a difference in survival (adjusted HR 0.84, 0.49 to 1.44; *P* = 0.536). In contrast, overall R1 resection was independently associated with worse OS (adjusted HR 1.82, 1.43 to 2.33; *P* < 0.001). Other independent predictors of OS included carbohydrate antigen 19-9, lymph node metastasis, tumour size ≥ 30 mm, and adjuvant chemotherapy. Year of surgery was not associated with survival. A sensitivity analysis with censored 90-day mortality yielded similar results (*Table S2*).

**Table 3:** Univariable and multivariable Cox regression analysis of overall survival for all patients who underwent DBD FS

	Univariable analysis		Multivariable analysis	
	Hazard ratio	*P*†	Hazard ratio	*P*‡
Age (years)*	1.00 (0.99, 1.01)	0.396		
**Sex**				
Female	1.00 (reference)			
Male	1.38 (0.91, 1.75)	0.410		
**Year of resection**				
2017–2023	1.00 (reference)		1.00 (reference)	
2010–2016	1.31 (1.03, 1.66)	0.022	1.01 (0.78, 1.30)	0.923
2003–2009	1.67 (1.18, 2.36)	0.003	1.23 (0.84, 1.79)	0.280
**ASA fitness grade**				
I	1.00 (reference)			
II	0.95 (0.72, 1.26)	0.758		
III	1.29 (0.93, 1.78)	0.122		
BMI (kg/m^2^)*	0.99 (0.97, 1.00)	0.342		
**Jaundice at presentation**				
No	1.00 (reference)		1.00 (reference)	
Yes	1.29 (1.02, 1.64)	0.028	1.24 (0.97, 1.59)	0.083
CA19-9 (units/ml)*	1.00 (1.00, 1.00)	0.002	1.00 (1.00, 1.00)	0.002
**Bismuth–Corlette type**				
I or II	1.00 (reference)		1.00 (reference)	
IIIa	1.69 (1.17, 2.44)	0.004	1.22 (0.82, 1.81)	0.322
IIIb	1.24 (0.84, 1.83)	0.271	1.08 (0.71, 1.65)	0.691
IV	1.57 (1.07, 2.31)	0.019	1.00 (0.66, 1.55)	0.971
**Operation type**				
Left sided hemihepatectomy	1.00 (reference)		1.00 (reference)	
Right sided hemihepatectomy	1.53 (1.23, 1.91)	< 0.001	1.27 (0.95, 1.70)	0.099
Central hepatectomy	1.51 (0.66, 3.42)	0.321	1.32 (0.57, 3.06)	0.505
**Tumour stage**				
< III	1.00 (reference)		1.00 (reference)	
≥ III	1.49 (1.20, 1.86)	< 0.001	1.17 (0.92, 1.50)	0.186
**Lymph node metastasis**				
No	1.00 (reference)		1.00 (reference)	
Yes	1.41 (1.14, 1.76)	0.001	1.46 (1.16, 1.83)	< 0.001
**Study group**				
Overall R0 after primary DBD FS	1.00 (reference)		1.00 (reference)	
Overall R0 after DBD re-resection or additional PD	0.91 (0.54, 1.54)	0.736	0.84 (0.49, 1.44)	0.536
Overall R1	1.66 (1.32, 2.08)	< 0.001	1.82 (1.43, 2.33)	< 0.001
**Perineural invasion**				
No	1.00 (reference)		1.00 (reference)	
Yes	1.34 (0.99, 1.83)	0.055	1.19 (0.87, 1.64)	0.263
**Tumour differentiation**				
Well	1.00 (reference)			
Moderate	0.92 (0.69, 1.24)	0.615		
Poor	1.09 (0.77, 1.77)	0.613		
**Tumour size (mm)**				
< 30	1.00 (reference)		1.00 (reference)	
≥ 30	1.81 (1.46, 2.25)	< 0.001	1.59 (1.26, 2.00)	< 0.001
**Adjuvant chemotherapy**				
No	1.00 (reference)		1.00 (reference)	
Yes	0.62 (0.49, 0.739	< 0.001	0.52 (0.40, 0.68)	< 0.001

Values in parentheses are 95% confidence intervals. *Hazard ratios shown per unit for continuous variables. The table includes imputed values based on 20 imputed data sets and 10 iterations. ASA, American Society of Anesthesiologists; BMI, body mass index; CA, carbohydrate antigen; DBD, distal bile duct; FS, frozen section; PD, pancreatoduodenectomy. †Significance at *P* < 0.100; ‡significance at *P* < 0.050.

In a stratified analysis, patients with overall R0 after DBD re-resection or additional PD had similar OS regardless of lymph node status. Median OS was 51.2 (27.0 to 69.5) months in the presence of lymph node metastasis compared with 55.2 (31.3 to 91.6) months in its absence (adjusted HR 0.90, 0.21 to 3.91; *P* = 0.896) (*Fig. S5*). No 90-day mortality was reported in this subgroup.

### Morbidity and mortality

Eleven patients undergoing additional PD were younger (by 19 years) than those undergoing DBD re-resection or no intervention (*P* = 0.013) and had better ASA grades (*P* = 0.040). No difference in Clavien–Dindo grade ≥ IIIa complications was found between groups (*P* = 0.126). ISGLS B or C bile leaks occurred in 3 patients (28%) who had additional PD, 11 (22%) who underwent DBD re-resection, and 116 (21.7%) who had no intervention (*P* = 0.674). Intra-abdominal abscess requiring drainage was more frequent in patients undergoing additional PD than in any other group (*P* = 0.022). The 90-day mortality rate did not differ between groups (*P* = 0.121), with no deaths reported among patients who had additional PD, 7 (14%) in the DBD re-resection group, and 49 (9.1%) among those with no intervention (*Table S3*). In the adjusted *post hoc* analysis, DBD re-resection was still not associated with increased risk of complications with Clavien–Dindo grade ≥ IIIa, ISLGS B or C bile leak, intra-abdominal abscess, or 90-day mortality (adjusted odds ratio 0.76 (95% c.i. 0.40 to 1.42), 1.07 (0.48 to 2.22), 0.40 (0.15 to 0.90), and 1.49 (0.55 to 3.59), respectively).

## Discussion

This was the first multicentre study to focus on intraoperative management of a positive DBD FS in pCCA^10^. Although only 45% of patients with a positive DBD FS achieved overall R0 status after DBD re-resection or additional PD, survival was better in these selected patients, irrespective of lymph node status. Morbidity and mortality did not differ between the groups.

Although DBD FS analysis was associated with conversion to overall R0 in a substantial subset of patients, its oncological impact appeared limited when considered in a broader clinical context. Technically, DBD resections can be extended more easily than PBD procedures following a positive FS. Yet, the present findings still align with those of Nooijen *et al.*^7^, who reported only a 5% increase in overall R0 rates following routine PBD FS analysis.

In this study, the overall R0 conversion rate was partly constrained by omitting any extended DBD resection in 27 of 73 patients with positive DBD margins. Concurrent involvement of the PBD margin and/or RM was also present in 35 of 73 patients. The risk of false-negative PBD FS has been well documented^7,34^ and invasive carcinoma at the RM dissection plane is often only detected on final pathology^27^. Furthermore, contrary to previous suggestions by Otsuka *et al.*^10,35^, intraoperative clearance of a positive DBD margin did not improve survival in patients with PBD or RM tumour spread (*Fig. 3*). These observations suggest that DBD re-resection or additional PD for a positive DBD margin should be carefully weighed in patients with suspected radial rather than longitudinal tumour growth. An alternative approach to improving patient selection could involve performing an upfront resection of the DBD deep into the pancreatic head, then waiting for the final pathology results before discussing the potential benefits and risks of an additional PD if necessary. Recent single-centre cohort studies^24–27,36,37^ from Eastern and Western institutions have also suggested individual association between a positive PBD margin or RM and survival. Thus, further research with larger cohorts is needed to determine whether a positive DBD margin also independently predicts worse oncological outcomes.

Given the large number of patients undergoing DBD FS analysis to determine eligibility for DBD re-resection or additional PD, it was crucial to determine whether these interventions conferred a meaningful survival advantage. The survival curve revealed that patients who achieved overall R0 after DBD re-resection or additional PD had comparable OS to those with overall R0 at primary DBD FS (*Fig. 2*). Conversely, patients with overall R1 status, irrespective of DBD re-resection or additional PD, experienced worse outcomes. These findings were verified by the multivariable analysis (*Table 3*), which revealed the independent prognostic value of achieving overall R0 following a positive DBD FS, even after adjusting for 90-day survival. Despite the well established prognostic role of lymph node metastasis^1,8,12,14,20,24–26,31,32^, overall R0 following DBD re-resection or additional PD was associated with similar survival irrespective of lymph node involvement. This should be interpreted with caution as the small sample size limited the power to exclude a clinically relevant effect.

In contrast to the present study, Otsuka *et al.*^10^ and Zhang *et al.*^11^ included patients with Bismuth–Corlette type I or II tumours treated with EHBD resection without major hepatectomy, likely contributing to the more favourable 90-day mortality and long-term outcomes. The overall R1 subgroup frequently included patients with Bismuth–Corlette type IIIa or IV tumours with larger lesion size and advanced T category, often necessitating complex right hepatectomies associated with greater operative risk and worse long-term prognosis^38^. Despite this, there was no difference in 90-day mortality between groups, and no early survival disparity appeared to drive any initial divergence between the survival curves. This discrepancy may reflect differences in tumour biology, patient selection, preoperative treatment, technical complexity or surgical strategies between Eastern and Western populations, as noted by Olthof *et al.*^1^ and Jansson *et al.*^2^. Nevertheless, a type II error cannot be ruled out owing to the limited sample size.

To evaluate the safety of extended resections, surgical morbidity and mortality were analysed. Patients who had DBD re-resection experienced rates of major surgical complications, bile leakage, and abscess formation similar to those of patients who did not undergo DBD re-resection, indicating that this procedure is generally safe. These results support the upfront use of the technique extending the DBD resection into the pancreatic head. Furthermore, additional PD was performed in younger patients with more favourable ASA grades and was not associated with 90-day mortality. Although Western series have previously reported high mortality rates following additional PD compared with Eastern cohorts^10,12,13,39^, this study emphasizes that the procedure may still be feasible in highly selected young and non-frail patients. However, broadening the indication to less selected populations may increase morbidity and mortality, and is unlikely to be justified in the absence of proven benefit.

This study had several limitations, including its retrospective design, variations in indications for adjuvant chemotherapy, and missing data on neoadjuvant therapy. The small sample size, together with the decision to perform DBD re-resection or additional PD at the operating surgeon’s discretion, introduced potential selection bias related to the identification of suitable candidates. A key limitation was potential interinstitutional heterogeneity in FS technique and margin reporting inherent to a retrospective multicentre design. This was mitigated by limiting participating centres to high-volume HPB centres, applying uniform ICCR definitions for margin status, and use of a standardized data file to register clearance of the margin site. Moreover, era-related variations in DBD FS outcomes did not independently affect survival after adjustment for co-variables. Nevertheless, a central pathology re-review was not undertaken, and variation in sampling or interpretation—particularly for HGD and subtle ductal changes—may persist. Although HGD was prespecified as negative, with comparable results in the sensitivity analysis that treated HGD as positive (*Fig. S4*), its prognostic significance remains uncertain and requires prospective validation, particularly given the small number of patients with HGD at the DBD margin (4 patients). The higher overall R0 rate after DBD re-resection or PD may reflect baseline differences, as these patients less commonly had Bismuth type IV tumours, and were more likely to have lower T categories and smaller tumours, consistent with more favourable biology. In addition, overall R0 status was more frequently achieved after right-sided resections, which are known to improve clearance of the PBD margin. These results suggest that underlying tumour biology combined with surgical strategy may have contributed to achieving overall R0 following DBD re-resection or PD. Therefore, a causal relationship between the technical achievement of overall R0 resection and improved prognosis cannot be established in this study. Additionally, the higher 90-day mortality rate in the overall R1 group (11.7%) may also reflect suboptimal selection in combination with frailty or co-morbidity, suggesting that resections were futile in a subset of patients. Another limitation is that indications and regimens for adjuvant chemotherapy varied between centres and over time, and patients who had overall R1 resections were more likely to have received treatment, introducing potential confounding. Although a randomized clinical trial would overcome these limitations, such a study is not feasible owing to the rarity of positive DBD FS in pCCA. Nevertheless, this remains the largest multicentre study providing real-world data reflecting clinical practice.

The present findings have indicated that extended DBD resection to achieve overall R0 status is associated with improved prognosis in selected patients, including those with lymph node metastasis. This strategy has a low risk of major complications following DBD re-resection, suggesting that routine extension of the resection into the pancreatic head may be performed safely—without the need for prior DBD FS—to optimize the likelihood of achieving negative margins. Additional PD proved safe when applied highly selectively.

## Supplementary Material

zrag010_Supplementary_Data

## Data Availability

The data sets from the present study are not publicly available owing to restrictions on ethical approval from the Danish Health Authority, Capital Region of Denmark (R-23057752). Data may be made available from the corresponding author upon reasonable request subject to necessary local approvals from each participating centre.
