# Supplementary material for: Clinical implications of an intraoperative margin-positive distal bile duct in perihilar cholangiocarcinoma: international multicentre cohort study
Source: BJS Open. 2026 Mar 23;10(2):zrag010. doi: 10.1093/bjsopen/zrag010 (PMC13008289; doi:10.1093/bjsopen/zrag010)
Supplement: zrag010_Supplementary_Data [file zrag010_supplementary_data.docx]

**Clinical implications of an intraoperative margin-positive distal bile duct in perihilar cholangiocarcinoma: international multicentre cohort study**

**Al-Saffar HA^1,2,3†^, ten Haaft BHEA^4,5†^, Marino R^6^, De Bellis M^7^, Olthof PB.^4,8^, Busund S^9^, Schultz NA^1^, Gilg S^3^, Ruzzenente A^7^, Koerkamp B^8^, Erdmann JI^4,5^, Ratti F^6^, Yaqub S^9^, Pommergaard HC^1,2,10^, Sturesson C^3^**

1. Department of Digestive Diseases, Transplantation and General Surgery, Centre for Cancer and Organ Diseases, Copenhagen University Hospital, Rigshospitalet, 2100 Copenhagen, Denmark.
2. Hepatic Malignancy Surgical Research Unit (HEPSURU), Copenhagen University Hospital, Rigshospitalet, 2100 Copenhagen, Denmark.
3. Division of Surgery and Oncology, Department of Clinical Science, Intervention and Technology, Karolinska Institutet and Karolinska University Hospital, 14152 Stockholm, Sweden.
4. Department of Surgery, Amsterdam UMC, University of Amsterdam, Amsterdam, The Netherlands.
5. Cancer Center Amsterdam, Amsterdam, The Netherlands
6. Hepatobiliary Surgery Division, IRCCS Ospedale San Raffaele, 20132 Milano, Italy.
7. Division of General and Hepatobiliary Surgery, Department of Surgery, Dentistry, Paediatrics and Gynaecology, G.B. Rossi Hospital, University of Verona, Verona, Italy.
8. Department of Surgery, Erasmus MC University Medical Center, 3015 Rotterdam, The Netherlands.
9. Department of Hepatopancreatobiliary Surgery, Oslo University Hospital, University of Oslo, N-0372 Oslo, Norway.
10. Department of Clinical Medicine, University of Copenhagen, 2100 Copenhagen, Denmark

**†** Al-Saffar HA and ten Haaft BHEA contributed equally to this work and are considered co–first authors

**Corresponding author:**

**Christian Sturesson**

Division of Surgery and Oncology, Department of Clinical Science, Intervention and Technology

Karolinska Institutet and Karolinska University Hospital

14152 Stockholm, Sweden. E-mail: [christian.sturesson@ki.se](mailto:christian.sturesson@ki.se)

During review process: **Hasan Ahmad Al-Saffar**

Department of Digestive Diseases, Transplantation and General Surgery, Centre for Cancer and Organ Diseases, Copenhagen University Hospital, Rigshospitalet.

2100 Copenhagen, Denmark. E-mail: [hasan.ahmad.al-saffar@regionh.dk](mailto:hasan.ahmad.al-saffar@regionh.dk)

**Supplementary Materials - Index**

| **Supplementary Results**  Reasons for omitting DBD FS  Survival data for the entire cohort (n=594)  **Supplementary Figures and Tables** | *pag. 2*  *pag. 2* |
| --- | --- |
| Fig. S1 | *pag. 2* |
| Fig. S2 | *pag. 3* |
| Fig. S3 | *pag. 3-4* |
| Table S1 | *pag. 4-5* |
| Table S2 | *pag. 5-6* |
| Table S3 | *pag. 6-7* |
| Fig. S4 | *pag. 7* |
| Table S4 | *pag. 7-8* |
| Table S5 | *pag. 8-9* |
| Table S6 | *pag. 9-10* |
| Table S7  Fig. S5 | *pag. 10*  *pag. 11* |
| **References** | *pag. 11-14* |

**Supplementary Results**

**Survival data for the entire cohort (n=594)**

After a median follow-up of 36.5 months, 304 patients (51.1 %) had recurrence, and 335 (56.4 %) had died. The median 1-, 3- and 5-year OS rates for the entire cohort were 82.9 % (95% c.i. 79.9—86.1), 50.9 % (95% c.i. 46.7—55.6) and 36.2 % (95% c.i. 31.7—41.2), respectively. DFS at 1-, 3- and 5-years was 77.4 % (95% c.i. 73.9—81.1), 43.6 % (95% c.i. 39.2—48.6) and 31.9 % (95% c.i. 27.3—37.3), respectively.

**Reasons for omitting DBD FS**

76 patients did not have an intraoperative DBD FS performed; reasons were discontinued practice of intraoperative FS in some centers (n=68), initial suspicion of colorectal liver metastasis or intrahepatic malignant stricture (n=2), prior DBD resection with existing anastomosis or hepatojejunostomy (n=2), surgeon’s preference not to perform routine intraoperative FS (n=1), selection due to frailty (1) or unknown reasons (n=2).

**Supplementary Figures and Tables**


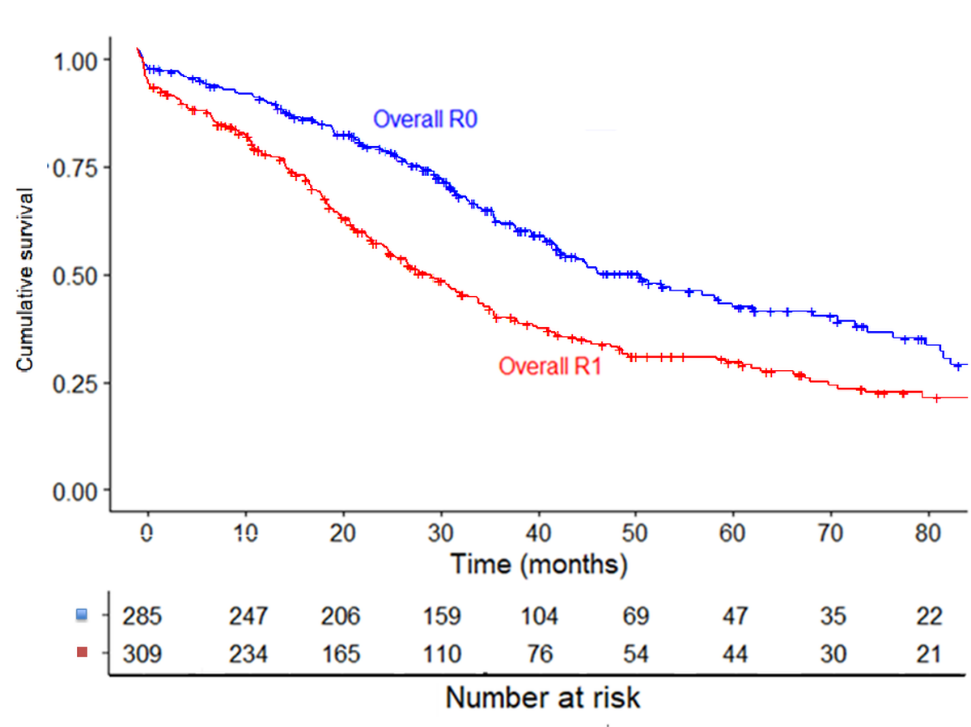


**Fig. S1:** Kaplan-Meier curve showing Overall R0 compared to Overall R1. Median overall survival was 47.1 months (26.6 to 99.9) compared to 28.1 months (14.5 to 67.8) respectively (P <0.001).


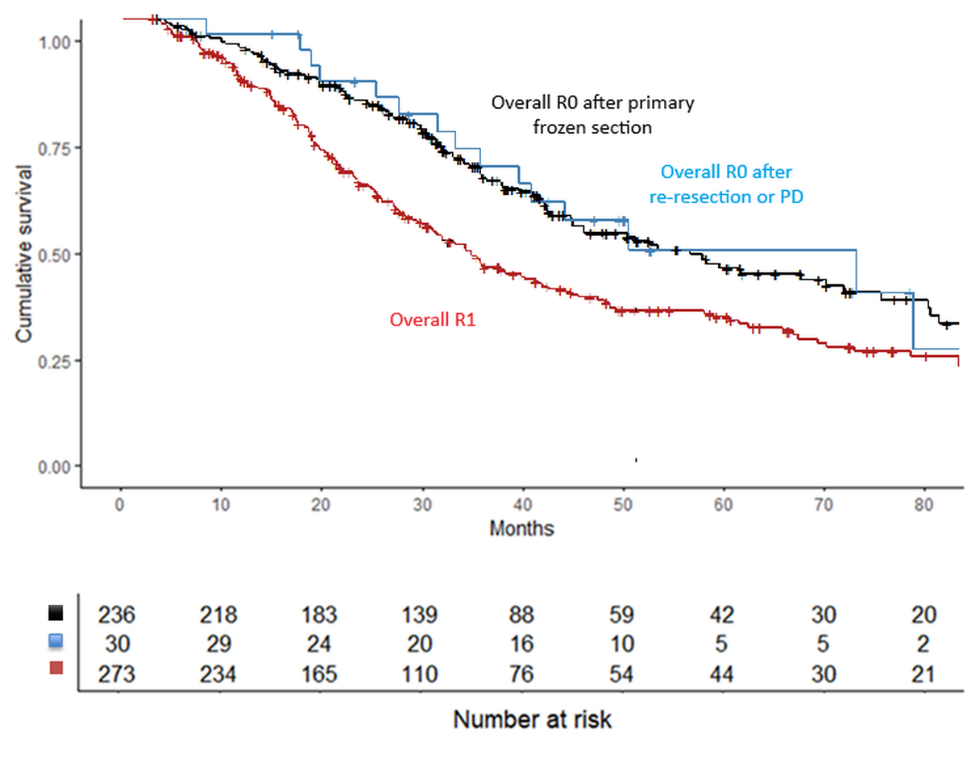


**Fig. S2:** Kaplan–Meier curve showing overall survival according to the outcome of DBD frozen section in patients surviving 90-days after resection. Patients were grouped as *Overall R0 after primary DBD frozen section, Overall R0 after re-resection of the DBD or additional PD*, and *Overall R1*. Median overall survival was 52.1 months (range: 30.0—81.2), 51.2 months (range: 31.2—80.4), and 32.3 months (range: 18.5—76.0), respectively. Pairwise comparisons are presented for median OS (*P* <0.05). Overall R0 after primary frozen section vs Overall R0 after re-resection or PD: *P* =0.968, Overall R0 after primary frozen section vs Overall R1: *P* <0.001. Overall R0 after re-resection or additional PD vs Overall R1: *P* =0.071.


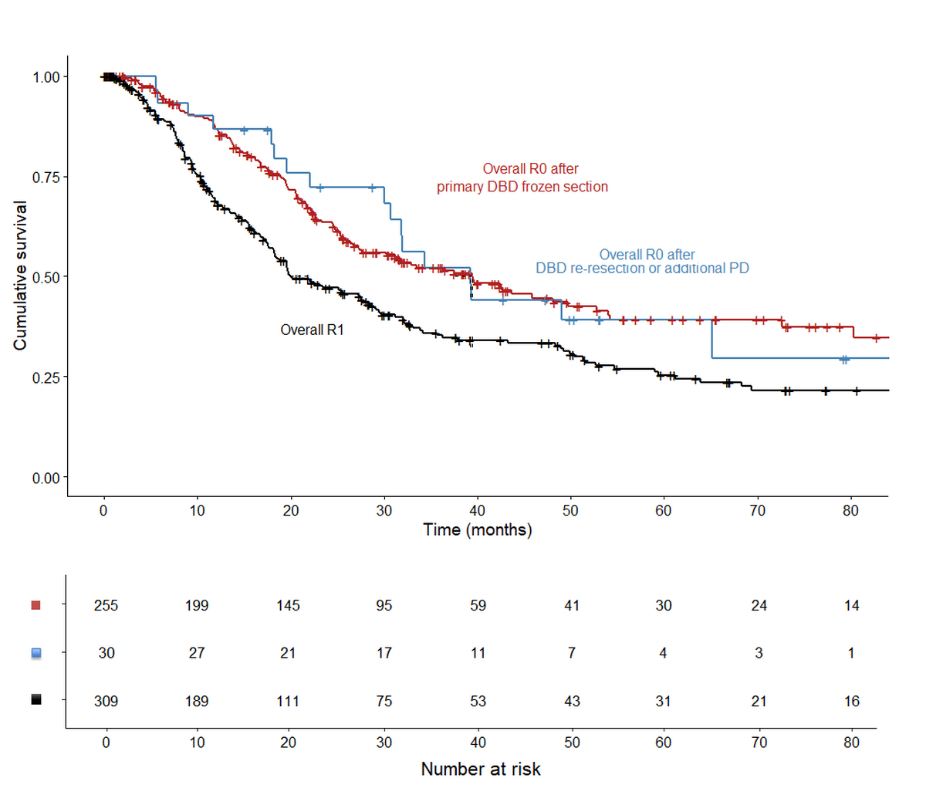


**Fig. S3:** Kaplan–Meier curve showing disease-free survival (DFS) according to the outcome of DBD frozen section. Patients were grouped *as Overall R0 after primary frozen section, Overall R0 after re-resection or additional PD*, and *Overall R1*. Median DFS was 39.3 months (18.8–71.2), 37.0 months (20.7–72.4), and 19.9 months (10.2–61.0), respectively. Pairwise comparisons are presented for median OS (*P* <0.050). Overall R0 after primary frozen section vs Overall R0 after re-resection or PD: *P* = 0.890. Overall R0 after primary frozen section vs Overall R1: *P* <0.001. Overall R0 after re-resection or PD vs Overall R1: *P* =0.060.

|  | **Univariable**  **HR (95% c.i.)** | ***P*-value^&^** | **Multivariable**  **HR (95% c.i.)** | ***P*-value^†^** |
| --- | --- | --- | --- | --- |
| Age (years) | 0.99 (0.98 to 0.99) | **0.037** | 0.99 (0.99 to 1.00) | 0.264 |
| Female (*ref*)  Male | 1  1.21 (0.91 to 1.58) | 0.105 |  |  |
| Year of surgery   - 2017 to 2023 - 2010 to 2016 - 2003 to 2009 | 1  1.14 (0.90 to 1.46)  1.09 (0.73 to 1.65) | 0.254  0.650 | 1  1.00 (0.78 to 1.29)  0.83 (0.54 to 1.28) |  |
| ASA  - 1 (*ref*)  - 2  - 3 | 1  0.85 (0.64 to 1.13)  0.91 (0.62 to 1.31) | 0.282  0.591 |  |  |
| BMI (kg/m^2^) | 0.98 (0.96 to 1.00) | 0.239 |  |  |
| No jaundice at presentation  Jaundice at presentation | 1  1.52 (1.18 to 1.95) | **0.001** | 1  1.54 (1.18 to 2.01) | **0.002** |
| CA 19-9 (U/ml) | 1.00 (0.99 to 1.00) | **0.009** | 1.00 (1.00 to 1.00) | **<0.001** |
| Bismuth-Corlett type   - I or II (*ref*) - IIIa - IIIb - IV | 1  1.21 (0.83 to 1.74)  1.22 (0.84 to 1.78)  1.51 (1.04 to 1.20) | 0.309  0.278  **0.028** | 1  0.84 (0.56 to 1.25)  1.27 (0.85 to 1.90)  1.09 (0.73 to 1.63) | 0.377  0.257  0.742 |
| Operati  on type   - Left sided hemihepatectomy (*ref*) - Right sided hemihepatectomy - Central hepatectomy | 1  1.29 (1.02 to 1.62)  1.67 (0.78 to 3.58) | **0.028**  0.182 | 1   \| 1.51 (1.13 to 2.00) \| \| --- \|   1.68 (0.77 to 3.71) | \| **0.005** \| \| --- \|   0.197 |
| Tumor stage <3 (*ref*)  Tumor stage ≥3 | 1  1.42 (1.13 to 1.78) | **0.002** | 1  1.12 (0.87 to 1.44) | 0.475 |
| No lymph node metastasis (*ref*)  Lymph node metastasis | 1  1.59 (1.27 to 2.00) | **<0.001** | 1  1.61 (1.27 to 2.05) | **<0.001** |
| Overall R0 after primary DBD frozen section (*ref*)  Overall R0 following DBD re-resection or additional PD^a^  Overall R1 | 1  0.98 (0.58 to 1.63)  1.60 (1.26 to 2.02) | 0.940  **<0.001** | 1  0.95 (0.56 to 1.61)  1.63 (1.27 to 2.09) | 0.913 **<0.001** |
| No perineural invasion (*ref*)  Perineural invasion | 1  1.47 (1.05 to 2.05) | **0.022** | 1  1.39 (0.98 to 1.95) | 0.060 |
| Tumor differentiation   - Well (*ref*) - Moderate - Poor | 1  1.02 (0.74 to 1.41)  1.11 (0.77 to 1.61) | 0.879  0.556 |  |  |
| Tumor size (mm)   - <30 (*ref*) - ≥30 | 1.72 (1.37 to 2.16) | **<0.001** | 1  1.46 (1.14 to 1.88) | **0.003** |
| No adjuvant chemotherapy (*ref*)  Adjuvant chemotherapy | 1  1.11 (0.88 to 1.41) | 0.348 |  |  |

**Table S1:** Uni- and multivariable Cox regression analysis for DFS (months) for all patients who underwent DBD frozen section. §: Censored in the multivariable analysis due to risk of multicollinearity. *Final pathological result in case of frozen section analysis with subsequent re-resection or PD. Statistically significant values in bold. &: Significance at P <0.100. **†**: Significance at P <0.050. Table includes imputed values based on 10 iterations and 20 imputations. *Ref*.: Reference category.

|  | **Univariable**  **HR (95% c.i.)** | ***P*-value^&^** | **Multivariable**  **HR (95% c.i.)** | ***P*-value^†^** |
| --- | --- | --- | --- | --- |
| Age (years) | 0.99 (0.99 to 1.00) | 0.969 |  |  |
| Female (*ref*)  Male | 1  1.30 (0.91 to 1.67) | 0.498 |  |  |
| Year of resection   - 2017 to 2023 (*ref*) - 2010 to 2016 - 2003 to 2009 | 1  1.19 (0.92 to 1.54)  1.50 (1.03 to 2.19) | 0.170  **0.032** | 0.32 (0.71 to 1.22)  1.10 (0.73 to 1.67) | 0.621  0.622 |
| ASA  - 1 (*ref*)  - 2  - 3 | 1  1.06 (0.81 to 1.53)  1.26 (0.87 to 1.93) | 0.680  0.213 |  |  |
| BMI (kg/m^2^) | 0.99 (0.97 to 1.00) | 0.320 |  |  |
| No jaundice at presentation  Jaundice at presentation | 1  1.27 (0.99 to 1.63) | **0.056** | 1.20 (0.92 to 1.56) | 0.177 |
| CA 19-9 (U/ml) | 1.00 (1.00 to 1.00) | **<0.001** | 1.00 (1.00 to 1.00) | **<0.001** |
| Bismuth-Corlett type   - I or II (*ref*) - IIIa - IIIb - IV | 1  1.65 (1.11 to 2.46)  1.30 (0.86 to 1.97)  1.58 (1.05 to 2.39) | **0.012**  0.208  **0.027** | 1.19 (0.70 to 1.82)  1.14 (0.72 to 1.78)  1.00 (0.64 to 1.56) | 0.427  0.565  0.974 |
| Operation type   - Left sided hemihepatectomy (*ref*) - Right sided hemihepatectomy - Central hepatectomy | 1  1.44 (1.14 to 1.83)  1.48 (0.60 to 3.62) | **0.001**  0.389 | 1  1.24 (0.91 to 1.70)  1.39 (0.55 to 3.48) | 0.159  0.478 |
| Tumor stage <3 (*ref*)  Tumor stage ≥3 | 1  1.62 (1.28 to 2.05) | **<0.001** | 1  1.28 (0.98 to 1.66) | 0.062 |
| No lymph node metastasis (*ref*)  Lymph node metastasis | 1  1.56 (1.24 to 1.97) | **<0.001** | 1  1.57 (1.23 to 2.01) | **<0.001** |
| Overall R0 after primary DBD frozen section (*ref*)  Overall R0 following DBD re-resection or additional PD^a^  Overall R1 | 1  1.02 (0.60 to 1.72)  1.64 (1.28 to 2.09) | 0.937  **<0.001** | 1  0.96 (0.56 to 1.66)  1.80 (1.38 to 2.34) | 0.909  **<0.001** |
| No perineural invasion (*ref*)  Perineural invasion | 1  1.43 (1.03 to 2.01) | **0.032** | 1.32 (0.93 to 1.86) | 0.116 |
| Tumor differentiation   - Well (*ref*) - Moderate - Poor | 1  0.95 (0.69 to 1.30)  1.05 (0.72 to 1.52) | 0.756  0.775 |  |  |
| Tumor size (mm)   - ≥30 (*ref*) - <30 | 1  1.87 (1.48 to 2.36) | **<0.001** | 1.65 (1.28 to 2.11) | **<0.001** |
| No adjuvant chemotherapy (*ref*)  Adjuvant chemotherapy | 1  0.74 (0.57 to 0.95) | **0.019** | 1  0.61 (0.46 to 0.81) | **<0.001** |

**Table S2:** Uni- and multivariable Cox regression with 90-day sensitivity analysis for OS (months) for all patients who underwent DBD frozen section. A: Pancreatoduodenectomy. Statistically significant values in bold. &: Significance at *P* <0.100. #: Significance at *P* <0.050. Table includes imputed values. *ref*.: Reference category.

| **Surgical outcome of DBD frozen section** | **Additional PD^a^ (n= 11)** | **DBD re-resection (n= 50*)** | **No DBD re-resection or additional PD (n=533)** | ***P*-value^†^** |
| --- | --- | --- | --- | --- |
| Age, median (IQR) | 50.0 (42.2-58.0) | 69.11 (74.94) | 67.39 (59.64-73.11) | **0.013^§^** |
| Sex, male  Missing: 0 | 8 (73) | 28 (56) | 329 (61.7) | 0.269^#^ |
| ASA  - 1  - 2  - 3  Missing: 0 | 2 (18)  8 (73)  1 (9) | 10 (20)  23 (46)  17 (34) | 129 (24.2)  294 (55.1)  110 (20.6) | **0.011^#^** |
| BMI, median (IQR)  Missing: 55 | 24.2 (22.0-26.8) | 23.0 | 24.3 | 0.374**^§^** |
| Bismuth-Corlett Type   - I - II - IIIa - IIIb - IV   Missing: 0 | 0 (0)  3 (27)  6 (55)  2 (18)  0 (0) | 6 (12)  7 (14)  16 (32)  7 (14)  14 (28) | 10 (1.8)  60 (11.2)  173 (32.4)  150 (28.1)  140 (26.2) | **<0.001^#^** |
| Preoperative biliary drainage   - ERCP - PTC - Both - None   Missing: 0 | 7 (64)  3 (27)  1 (9)  0 (0) | 14 (28)  26 (52)  3 (6)  7 (14) | 153 (28.7)  204 (38.2)  66 (12.3)  110 (20.6) | **<0.001^#^** |
| Portal vein embolization, n (%)  Missing: 0 | 2 (18) | 14 (28) | 105 (19.6) | 0.158^#^ |
| Right sided hemihepatectomy  Left sided hemihepatectomy  Central hepatectomy  Missing: 0 | 8 (73)  3 (27)  0 (0) | 33 (66)  15 (30)  2 (4) | 253 (47.4)  271 (50.8)  9 (1.6) | **<0.001^#^** |
| Vascular reconstruction, n (%)  Missing: 0 | 1 (9) | 15 (30) | 113 (21.1) | 0.075^#^ |
| CD^b^ 3a or more  Missing: 6 | 8 (73) | 24 (48) | 264 (49.5) | 0.126^#^ |
| Bile leakage grade B or C  Missing: 6 | 3 (27) | 11 (22) | 116 (21.7) | 0.674^#^ |
| Postoperative intraabdominal abscess (drained)  Missing: 3 | 4 (36) | 6 (12) | 134 (25.1) | **0.022^#^** |
| 90-day mortality | 0 (0) | 7 (14) | 49 (9.1) | 0.121^#^ |

**Table S3:** Complication features of patients with positive DBD frozen section according to surgical management. a: Pancreatoduodenectomy. b: Clavien-Dindo. #, Chi-Square test; §, Kruskal-Wallis test. All values are described as n (%) unless stated otherwise. *One patient underwent re-resection due to inconclusive result with benign re-resection and pathology at re-examination. †: Significance at *P* <0.050. Significant values in bold. Due to rounding, percentages may not total 100.


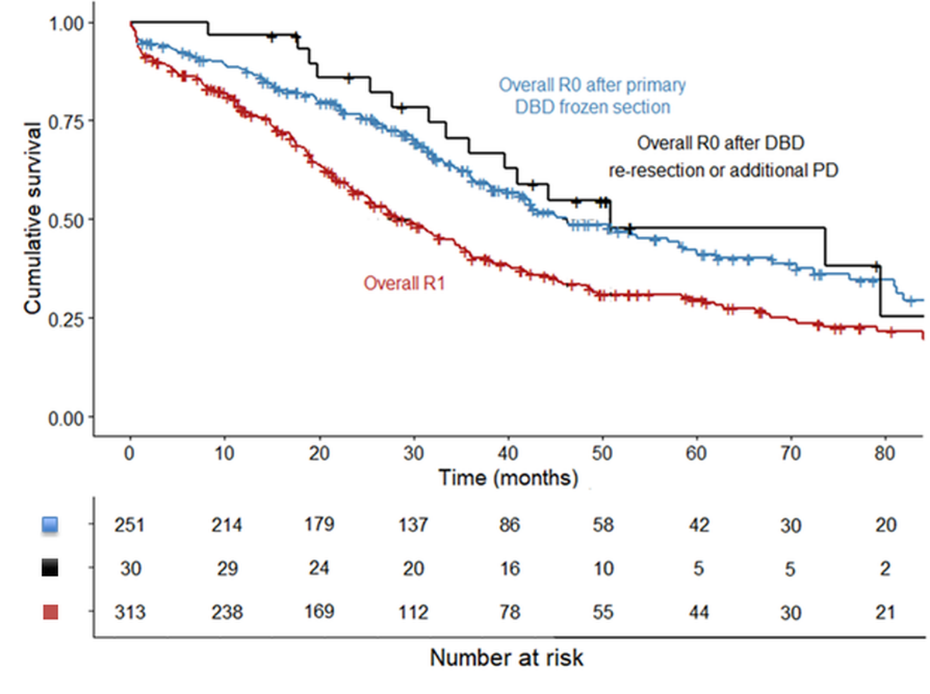


**Fig. S4:** Kaplan–Meier curve showing overall survival according to the outcome of DBD frozen section, with HGD defined as positive. Patients were grouped as overall R0 after primary DBD frozen section, overall R0 after DBD re-resection or additional PD, and overall R1. Median OS was 45.6 months (range: 25.0—85.2), 51.2 months (range: 31.2—80.4), and 28.6 months (range: 14.6—68.2), respectively. Pairwise comparisons are presented for median OS (*P* <0.05). Overall R0 after primary frozen section vs overall R0 after re-resection or PD: *P* = 1.000. Overall R0 after primary frozen section vs overall R1: *P* <0.001. Overall R0 after re-resection or additional PD vs overall R1: *P* =0.070

|  | **Univariable**  **OR (95% c.i.)** | ***P*-value^&^** | **Multivariable**  **aOR (95% c.i.)** | ***P*-value^†^** |
| --- | --- | --- | --- | --- |
| Age (years) | 0.99 (0.98 to 1.01) | 0.596 | 1.05 (0.98 to 1.02) | 0.575 |
| Female (*ref*)  Male | 1  0.92 (0.66 to 1.29) | 0.646 |  |  |
| Year of resection   - 2017 to 2023 (*ref*) - 2010 to 2016 - 2003 to 2009 | 1  1.59 (1.12 to 2.26)  0.92 (0.50 to 1.68) | **0.009**  0.798 | 1  1.23 (0.82 to 1.83)  0.87 (0.43 to 1.72) | 0.304  0.694 |
| ASA  - 1 (*ref*)  - 2  - 3 | 1  1.28 (0.86 to 1.92)  1.43 (0.88 to 2.33) | 0.221  **0.144** | 1  1.30 (0.83 to 2.02)  1.52 (0.89 to 2.61) | 0.242  0.126 |
| BMI (kg/m^2^) | 0.99 (0.96 to 1.00) | 0.346 |  |  |
| No jaundice at presentation  Jaundice at presentation | 1  0.58 (0.40 to 0.83) | **0.003** | 1  0.42 (0.27 to 0.65) | **<0.001** |
| Bismuth-Corlett type   - I or II (*ref*) - IIIa - IIIb - IV | 1  2.51 (1.49 to 4.30)  1.13 (0.66 to 1.96)  1.34 (0.78 to 2.32) | **<0.001**  0.645  0.285 | 1  1.57 (0.88 to 2.81)  1.38 (0.74 to 2.57)  1.26 (0.70 to 2.28) | 0.121  0.304  0.435 |
| Tumor size (mm) | 1.01 (1.00 to 1.02) | **0.010** | 1.01 (0.99 to 1.02) | 0.078 |
| Preoperative endoscopic decompression   - None (*ref*) - ERCP - PTC - Both | 1  1.83 (1.16 to 2.91)  2.01 (1.24 to 3.29)  3.26 (1.74 to 6.21) | **0.009**  **0.004**  **<0.001** | 1  2.54 (1.50 to 4.39)  2.47 (1.42 to 4.35)  3.92 (1.92 to 8.21) | **<0.001**  **0.001**  **<0.001** |
| Operation type   - Left sided hemihepatectomy (*ref*) - Right sided hemihepatectomy - Central hepatectomy | 1  2.10 (1.50 to 2.94)  0.53 (0.11 to 1.89) | **<0.001**  0.365 | 1  1.87 (1.19 to 2.95)  0.78 (0.16 to 2.92) | **0.006**  0.735 |
| No vascular resection (*ref*)  Vascular resection | 1  0.91 (0.54 to 1.54) | 0.736 | 1  0.84 (0.49 to 1.44) | 0.536 |
| No DBD re-resection (*ref*)  DBD re-resection | 1  0.92 (0.51 to 1.64) | 0.777 | 1  0.76 (0.40 to 1.42) | 0.396 |

**Table S4:** Logistic regression analysis with Clavien-Dindo 3a or more (CD ≥3) as outcome, in all patients who underwent distal bile duct (DBD) frozen section; Patients who underwent Pancreatoduodenectomy were excluded from the analysis. Statistically significant values in bold. &: Significance at *P* <0.200; Variables of clinical relevance are included in the multivariable analysis. #: Significance at *P* <0.050. Table includes imputed values. *ref*.: Reference category.

|  | **Univariable**  **OR (95% c.i.)** | ***P*-value^&^** | **Multivariable**  **aOR (95% c.i.)** | ***P*-value^†^** |
| --- | --- | --- | --- | --- |
| Age (years) | 1.00 (0.98 to 1.02) | 0.814 |  |  |
| Female (*ref*)  Male | 1  1.11 (0.74 to 1.68) | 0.603 |  |  |
| Year of resection   - 2017 to 2023 (*ref*) - 2010 to 2016 - 2003 to 2009 | 1  1.37 (0.90 to 2.08)  1.29 (0.61 to 2.54) | **0.141**  0.481 | 1  1.22 (0.77 to 1.91)  1.08 (0.47 to 2.33) | 0.390  0.856 |
| ASA  - 1 (*ref*)  - 2  - 3 | 1  1.12 (0.70 to 1.84)  0.75 (0.40 to 1.37) | 0.634  0.351 | 1  1.17 (0.71 to 1.97)  0.85 (0.44 to 1.63) | 0.545  0.632 |
| BMI (kg/m^2^) | 1.00 (0.99 to 1.02) | 0.512 |  |  |
| No jaundice at presentation  Jaundice at presentation | 1  0.72 (0.48 to 1.10) | **0.126** | 1  0.60 (0.38 to 0.96) | **0.033** |
| Bismuth-Corlett type   - I or II (*ref*) - IIIa - IIIb - IV | 1  1.12 (0.60 to 2.20)  1.01 (0.52 to 2.03)  1.56 (0.82 to 3.06) | 0.723  0.970  **0.181** | 1  1.24 (0.62 to 2.59)  0.67 (0.32 to 1.43)  1.20 (0.60 to 2.46) | 0.557  0.294  0.614 |
| Tumor size (mm) | 1.01 (1.00 to 1.02) | **0.002** | 1.016 (1.00 to 1.02) | **0.004** |
| Preoperative endoscopic decompression   - None (*ref*) - ERCP - PTC - Both | 1  1.24 (0.72 to 2.20)  1.37 (0.77 to 2.49)  1.19 (0.55 to 2.49) | 0.455  0.290  0.657 | 1  1.23 (0.67 to 2.31)  1.43 (0.76 to 2.74)  1.31 (0.57 to 2.97) | 0.511  0.272  0.515 |
| Operation type   - Left sided hemihepatectomy (*ref*) - Right sided hemihepatectomy - Central hepatectomy | 1  0.71 (0.47 to 1.06)  5.51 (1.61 to 21.56) | **0.092**  **0.007** | 1  0.50 (0.29 to 0.85)  5.72 (1.60 to 23.27) | **0.011**  **0.008** |
| No vascular resection (*ref*)  Vascular resection | 1  1.28 (0.80 to 2.01) | 0.300 |  |  |
| No distal bile duct re-resection (*ref*)  Distal bile duct re-resection | 1  1.00 (0.47 to 1.95) | 0.999 | 1  1.07 (0.48 to 2.22) | 0.862 |

**Table S5:** Logistic regression analysis with ISGLS bile leakage grade B or C as outcome, in all patients who underwent distal bile duct (DBD) frozen section; Patients who underwent Pancreatoduodenectomy were excluded from the analysis. Statistically significant values in bold. &: Significance at *P* <0.200; Variables of clinical relevance are included in the multivariable analysis. #: Significance at *P* <0.050. Table includes imputed values. *ref*.: Reference category.

|  | **Univariable**  **OR (95% c.i.)** | ***P*-value^&^** | **Multivariable**  **aOR (95% c.i.)** | ***P*-value^†^** |
| --- | --- | --- | --- | --- |
| Age (years) | 1.00 (0.99 to 1.02) | 0.694 |  |  |
| Female (*ref*)  Male | 1  1.06 (0.72 to 1.58) | 0.758 |  |  |
| Year of resection   - 2017 to 2023 (*ref*) - 2010 to 2016 - 2003 to 2009 | 1  1.60 (1.08 to 2.38)  0.40 (0.13 to 0.95) | **0.020**  0.060 | 1  1.28 (0.84 to 1.95)  0.26 (0.08 to 0.65) | 0.253  **0.008** |
| ASA  - 1 (*ref*)  - 2  - 3 | 1  1.09 (0.69 to 1.75)  0.68 (0.37 to 1.22) | 0.702  **0.198** | 1  1.18 (0.73 to 1.92)  0.74 (0.39 to 1.37) | 0.512  0.336 |
| BMI (kg/m^2^) | 0.98 (0.93 to 1.01) | 0.466 |  |  |
| No jaundice at presentation  Jaundice at presentation | 1  0.87 (0.58 to 1.31) | 0.490 |  |  |
| Bismuth-Corlett type   - I or II (*ref*) - IIIa - IIIb - IV | 1  1.21 (0.67 to 2.26)  1.13 (0.61 to 2.15)  0.89 (0.47 to 1.72) | 0.534  0.698  0.725 |  |  |
| Tumor size (mm) | 1.01 (1.00 to 1.02) | **0.080** | 1.01 (1.00 to 1.02) | 0.123 |
| Preoperative endoscopic decompression   - None (*ref*) - ERCP - PTC - Both | 1  0.97 (0.58 to 1.67)  1.02 (0.58 to 1.79)  1.67 (0.85 to 3.25) | 0.922  0.957  **0.134** | 1  0.89 (0.52 to 1.54)  0.83 (0.46 to 1.49)  1.28 (0.63 to 2.60) | 0.667  0.526  0.495 |
| Operation type   - Left sided hemihepatectomy (*ref*) - Right sided hemihepatectomy - Central hepatectomy | 1  1.46 (1.00 to 2.16)  0.85 (0.13 to 3.40) | **0.054**  0.835 | 1  1.54 (1.03 to 2.31)  1.23 (0.18 to 5.18) | **0.036**  0.803 |
| No vascular resection (*ref*)  Vascular resection | 1  1.62 (1.04 to 2.50) | **0.029** | 1  1.47 (0.91 to 2.34) | 0.107 |
| No distal bile duct re-resection (*ref*)  Distal bile duct re-resection | 1  0.40 (0.15 to 0.90) | **0.042** | 1  0.40 (0.15 to 0.90) | **0.043** |

**Table S6:** Logistic regression analysis with intra-abdominal abscess as outcome, in all patients who underwent distal bile duct (DBD) frozen section; Patients who underwent Pancreatoduodenectomy were excluded from the analysis. Statistically significant values in bold. &: Significance at *P* <0.200; Variables of clinical relevance are included in the multivariable analysis. #: Significance at *P* <0.050. Table includes imputed values. *ref*.: Reference category.

|  | **Univariable**  **OR (95% c.i.)** | ***P*-value^&^** | **Multivariable**  **aOR (95% c.i.)** | ***P*-value^†^** |
| --- | --- | --- | --- | --- |
| Age (years) | 1.03 (1.01 to 1.07) | **0.012** | 1.03 (1.00 to 1.07) | **0.015** |
| Female (*ref*)  Male | 1  1.77 (0.87 to 3.39) | 0.269 |  |  |
| Year of resection   - 2017 to 2023 (*ref*) - 2010 to 2016 - 2003 to 2009 | 1  1.56 (0.86 to 2.84)  2.00 (0.76 to 4.73) | 1  **0.139**  **0.129** | 1  1.39 (0.71 to 2.69)  1.45 (0.47 to 4.01) | 0.325  0.485 |
| ASA  - 1 (*ref*)  - 2  - 3 | 1  0.42 (0.21 to 0.85)  1.11 (0.54 to 2.25) | **0.014**  0.770 | 1  0.50 (0.24 to 1.02)  1.20 (0.56 to 2.58) | 0.057  0.632 |
| BMI (kg/m^2^) | 0.99 (0.93 to 1.01) | 0.567 |  |  |
| No jaundice at presentation  Jaundice at presentation | 1  1.48 (0.79 to 2.94) | 0.235 |  |  |
| Bismuth-Corlett type   - I or II (*ref*) - IIIa - IIIb - IV | 1  1.86 (0.77 to 5.20)  0.78 (0.27 to 2.40)  1.48 (0.58 to 4.28) | **0.191**  0.649  0.426 | 1  1.50 (0.57 to 4.48)  0.63 (0.18 to 2.25)  1.21 (0.44 to 3.68) | 0.426  0.465  0.717 |
| Tumor size (mm) | 1.01 (0.99 to 1.02) | **0.051** | 1.00 (0.99 to 1.02) | 0.257 |
| Preoperative endoscopic decompression   - None (*ref*) - ERCP - PTC - Both | 1  0.78 (0.37 to 1.72)  0.67 (0.29 to 1.58)  1.84 (0.77 to 4.40) | 0.539  0.362  **0.165** | 1  0.66 (0.29 to 1.54)  0.66 (0.26 to 1.63)  1.29 (0.48 to 3.41) | 0.335  0.367  0.596 |
| Operation type   - Left sided hemihepatectomy (*ref*) - Right sided hemihepatectomy - Central hepatectomy | 1  1.96 (1.10 to 3.57)  1.40 (0.07 to 7.93) | **0.024**  0.751 | 1  1.22 (0.56 to 2.68)  1.10 (0.05 to 6.94) | 0.613  0.929 |
| No vascular resection (*ref*)  Vascular resection | 1  2.43 (1.33 to 4.32) | **0.002** | 1  2.01 (1.05 to 3.76) | **0.031** |
| No distal bile duct re-resection (*ref*)  Distal bile duct re-resection | 1  1.64 (0.64 to 3.65) | 0.252 | 1  1.49 (0.55 to 3.59) | 0.396 |

**Table S7:** Logistic regression analysis with 90-day mortality as outcome, in all patients who underwent distal bile duct (DBD) frozen section; Patients who underwent Pancreatoduodenectomy were excluded from the analysis. Statistically significant values in bold. &: Significance at *P* <0.200; Variables of clinical relevance are included in the multivariable analysis. #: Significance at *P* <0.050. Table includes imputed values. *ref*.: Reference category.


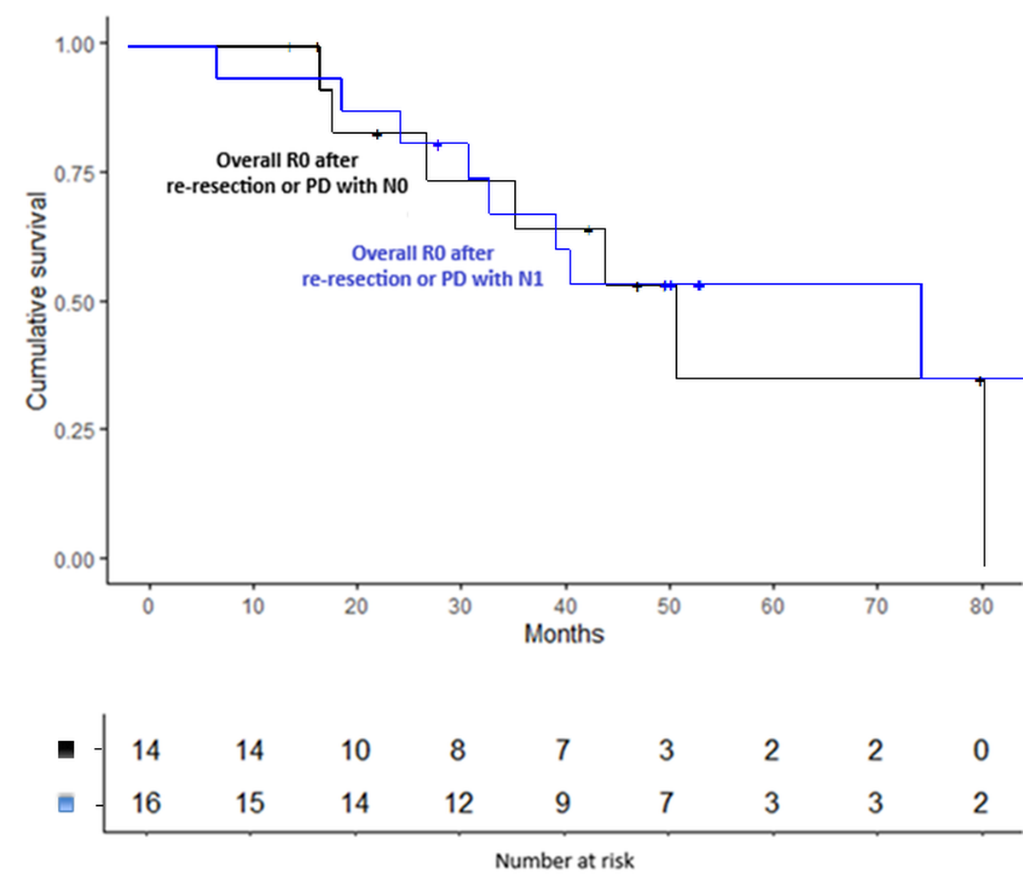


**Fig. S5:** Kaplan-Meier curve showing overall R0 after re-resection or PD without lymph node metastasis (N0) vs Overall R0 after re-resection or additional PD with lymph node metastasis (N1 or N2). Median overall survival was 51.2 months (95% c.i. 27.0—69.5) compared to 55.2 months (95% c.i. 31.3—91.6), P=0.670.

**References**

1. Olthof PB, Miyasaka M, Koerkamp BG, Wiggers JK, Jarnagin WR, Noji T, et al. A comparison of treatment and outcomes of perihilar cholangiocarcinoma between Eastern and Western centers. HPB. 2019 Mar;21(3):345–51.

2. Jansson H, Oba A, Maekawa A, Villard C, Kobayashi K, Ono Y, et al. Western and Eastern experience in treating perihilar cholangiocarcinoma: retrospective bi-centre study. BJS Open. 2025 Mar 4;9(2).

3. Mueller M, Breuer E, Mizuno T, Bartsch F, Ratti F, Benzing C, et al. Perihilar Cholangiocarcinoma – Novel Benchmark Values for Surgical and Oncological Outcomes From 24 Expert Centers. Ann Surg. 2021 Nov;274(5):780–8.

4. Rassam F, Roos E, van Lienden KP, van Hooft JE, Klümpen HJ, van Tienhoven G, et al. Modern work-up and extended resection in perihilar cholangiocarcinoma: the AMC experience. Langenbecks Arch Surg. 2018 May 19;403(3):289–307.

5. Sturesson C, Soreide K. Preoperative management and resectability criteria in perihilar cholangiocarcinoma. British Journal of Surgery. 2025 Mar 28;112(4).

6. Nagino M, DeMatteo R, Lang H, Cherqui D, Malago M, Kawakatsu S, et al. Proposal of a New Comprehensive Notation for Hepatectomy. Ann Surg. 2021 Jul;274(1):1–3.

7. Nooijen LE, Franken LC, de Boer MT, Buttner S, van Dieren S, Koerkamp BG, et al. Value of routine intraoperative frozen sections of proximal bile duct margins in perihilar cholangiocarcinoma, a retrospective multicenter and matched case-control study. European Journal of Surgical Oncology. 2022 Dec;48(12):2424–31.

8. Endo I, House MG, Klimstra DS, Gönen M, D’Angelica M, DeMatteo RP, et al. Clinical Significance of Intraoperative Bile Duct Margin Assessment for Hilar Cholangiocarcinoma. Ann Surg Oncol. 2008 Aug 10;15(8):2104–12.

9. Higuchi R, Yazawa T, Uemura S, Izumo W, Furukawa T, Yamamoto M. High‐grade dysplasia/carcinoma *in situ* of the bile duct margin in patients with surgically resected node‐negative perihilar cholangiocarcinoma is associated with poor survival: a retrospective study. J Hepatobiliary Pancreat Sci. 2017 Aug 3;24(8):456–65.

10. Otsuka S, Ebata T, Yokoyama Y, Mizuno T, Tsukahara T, Shimoyama Y, et al. Clinical value of additional resection of a margin-positive distal bile duct in perihilar cholangiocarcinoma. British Journal of Surgery. 2019 Apr 11;106(6):774–82.

11. Zhang XF, Squires MH, Bagante F, Ethun CG, Salem A, Weber SM, et al. The Impact of Intraoperative Re-Resection of a Positive Bile Duct Margin on Clinical Outcomes for Hilar Cholangiocarcinoma. Ann Surg Oncol. 2018 May 22;25(5):1140–9.

12. D’Souza MA, Valdimarsson VT, Campagnaro T, Cauchy F, Chatzizacharias NA, D’Hondt M, et al. Hepatopancreatoduodenectomy –a controversial treatment for bile duct and gallbladder cancer from a European perspective. HPB. 2020 Sep;22(9):1339–48.

13. Tran TB, Dua MM, Spain DA, Visser BC, Norton JA, Poultsides GA. Hepato-pancreatectomy: how morbid? Results from the national surgical quality improvement project. HPB. 2015 Sep;17(9):763–9.

14. Koerkamp BG, Wiggers JK, Allen PJ, Besselink MG, Blumgart LH, Busch ORC, et al. Recurrence Rate and Pattern of Perihilar Cholangiocarcinoma after Curative Intent Resection. J Am Coll Surg. 2015 Dec;221(6):1041–9.

15. Elshami M, Hue JJ, Ahmed FA, Kakish H, Hoehn RS, Rothermel LD, et al. Defining Facility Volume Threshold for Optimization of Short- and Long-Term Outcomes in Patients Undergoing Resection of Perihilar Cholangiocarcinoma. Journal of Gastrointestinal Surgery. 2023 Apr;27(4):730–40.

16. Olthof PB, Bouwense SAW, Bednarsch J, Dewulf M, Kazemier G, Maithel S, et al. Failure to Rescue After Resection of Perhilar Cholangiocarcinoma in an International Multicenter Cohort. Ann Surg Oncol. 2025 Mar 15;32(3):1762–8.

17. von Elm E, Altman DG, Egger M, Pocock SJ, Gøtzsche PC, Vandenbroucke JP. The Strengthening the Reporting of Observational Studies in Epidemiology (STROBE) statement: guidelines for reporting observational studies. J Clin Epidemiol. 2008 Apr;61(4):344–9.

18. Hendrix JM, Garmon EH. American Society of Anesthesiologists Physical Status Classification System. 2025.

19. D’Souza MA, Al-Saffar HA, Fernández Moro C, Shtembari S, Danielsson O, Sparrelid E, et al. Redefining resection margins and dissection planes in perihilar cholangiocarcinoma—radical resection is a rare event. Virchows Archiv. 2022 Mar 16;480(3):557–64.

20. Yamamoto R, Onoe S, Mizuno T, Watanabe N, Kawakatsu S, Sunagawa M, et al. Reappraisal of carcinoma in situ residue at the bile duct margin: a single-center review of 681 patients with perihilar cholangiocarcinoma. HPB. 2025 Mar;27(3):362–70.

21. Igami T, Nagino M, Oda K, Nishio H, Ebata T, Yokoyama Y, et al. Clinicopathologic Study of Cholangiocarcinoma With Superficial Spread. Ann Surg. 2009 Feb;249(2):296–302.

22. Amin MB, Greene FL, Edge SB, Compton CC, Gershenwald JE, Brookland RK, et al. The Eighth Edition <scp>AJCC</scp> Cancer Staging Manual: Continuing to build a bridge from a population‐based to a more “personalized” approach to cancer staging. CA Cancer J Clin. 2017 Mar 17;67(2):93–9.

23. Burt AD, Alves V, Bedossa P, Clouston A, Guido M, Hübscher S, et al. Data set for the reporting of intrahepatic cholangiocarcinoma, perihilar cholangiocarcinoma and hepatocellular carcinoma: recommendations from the International Collaboration on Cancer Reporting (ICCR). Histopathology. 2018 Sep 30;73(3):369–85.

24. Ratti F, Marino R, Pedica F, Gardini AC, Cipriani F, Rimini M, et al. Radial and longitudinal margins in surgery of perihilar cholangiocarcinoma: When R1 definition is associated with different prognosis. Surgery. 2023 Sep;174(3):447–56.

25. De Bellis M, Mastrosimini MG, Conci S, Pecori S, Campagnaro T, Castelli C, et al. The Prognostic Role of True Radical Resection in Perihilar Cholangiocarcinoma after Improved Evaluation of Radial Margin Status. Cancers (Basel). 2022 Dec 12;14(24):6126.

26. Shinohara K, Ebata T, Shimoyama Y, Mizuno T, Yokoyama Y, Yamaguchi J, et al. A Study on Radial Margin Status in Resected Perihilar Cholangiocarcinoma. Ann Surg. 2021 Mar;273(3):572–8.

27. D’Amico FE, Mescoli C, Caregari S, Pasquale A, Billato I, Alessandris R, et al. Impact of Positive Radial Margin on Recurrence and Survival in Perihilar Cholangiocarcinoma. Cancers (Basel). 2022 Mar 25;14(7):1680.

28. Dindo D, Demartines N, Clavien PA. Classification of Surgical Complications. Ann Surg. 2004 Aug;240(2):205–13.

29. Koch M, Garden OJ, Padbury R, Rahbari NN, Adam R, Capussotti L, et al. Bile leakage after hepatobiliary and pancreatic surgery: A definition and grading of severity by the International Study Group of Liver Surgery. Surgery. 2011 May;149(5):680–8.

30. McShane LM, Altman DG, Sauerbrei W, Taube SE, Gion M, Clark GM, et al. Reporting recommendations for tumor marker prognostic studies (REMARK). J Natl Cancer Inst [Internet]. 2005 [cited 2025 Sep 28];97(16):1180–4. Available from: https://pubmed.ncbi.nlm.nih.gov/16106022/

31. Groot Koerkamp B, Wiggers JK, Gonen M, Doussot A, Allen PJ, Besselink MGH, et al. Survival after resection of perihilar cholangiocarcinoma—development and external validation of a prognostic nomogram. Annals of Oncology. 2015 Sep;26(9):1930–5.

32. Ratti F, Marino R, Olthof PB, Pratschke J, Erdmann JI, Neumann UP, et al. Predicting futility of upfront surgery in perihilar cholangiocarcinoma: Machine learning analytics model to optimize treatment allocation. Hepatology. 2024 Feb;79(2):341–54.

33. van Keulen AM, Buettner S, Erdmann JI, Pratschke J, Ratti F, Jarnagin WR, et al. Multivariable prediction model for both 90-day mortality and long-term survival for individual patients with perihilar cholangiocarcinoma: does the predicted survival justify the surgical risk? British Journal of Surgery. 2023 Apr 12;110(5):599–605.

34. Mantel HTJ, Westerkamp AC, Sieders E, Peeters PMJG, de Jong KP, Boer MT, et al. Intraoperative frozen section analysis of the proximal bile ducts in hilar cholangiocarcinoma is of limited value. Cancer Med. 2016 Jul 6;5(7):1373–80.

35. Otsuka S, Nagino M. Author response to: Comment on Clinical value of additional resection of a margin-positive distal bile duct in perihilar cholangiocarcinoma. British Journal of Surgery. 2019 Jul 15;106(9):1258–9.

36. Jin S, Lin MY, Xiang CH, Liu ZP, Wang SY, Jiang N, et al. The Status of Liver Margin in Perihilar Cholangiocarcinoma. Ann Surg. 2024 Oct 21;

37. ten Haaft BHEA, Al-Saffar HA, Roos E. Prognostic Impact of Individual Resection and Dissection Margins in Resected Perihilar Cholangiocarcinoma . Unpublished data.

38. Olthof PB, Erdmann JI, Alikhanov R, Charco R, Guglielmi A, Hagendoorn J, et al. Higher Postoperative Mortality and Inferior Survival After Right-Sided Liver Resection for Perihilar Cholangiocarcinoma: Left-Sided Resection is Preferred When Possible. Ann Surg Oncol. 2024 Jul 12;31(7):4405–12.

39. Kawano F, Yoshioka R, Ichida H, Mise Y, Saiura A. Essential updates 2021/2022: Update in surgical strategy for perihilar cholangiocarcinoma. Ann Gastroenterol Surg. 2023 Nov 8;7(6):848–55.
